# Deriving meaning from others’ emotions: attribution, appraisal, and the use of emotions as social information

**DOI:** 10.3389/fpsyg.2015.01077

**Published:** 2015-07-28

**Authors:** Evert A. van Doorn, Gerben A. van Kleef, Joop van der Pligt

**Affiliations:** Department of Social Psychology, University of AmsterdamAmsterdam, Netherlands

**Keywords:** emotion, emotional expression, attribution, social appraisal, emotions as social information, sense-making

## Abstract

Emotional expressions constitute a rich source of information. Integrating theorizing on attribution, appraisal processes, and the use of emotions as social information, we examined how emotional expressions influence attributions of agency and responsibility under conditions of ambiguity. Three vignette studies involving different scenarios indicate that participants used information about others’ emotional expressions to make sense of ambiguous social situations. Expressions of regret fueled inferences that the expresser was responsible for an adverse situation, whereas expressions of anger fueled inferences that someone else was responsible. Also, expressions of anger were interpreted as a sign of injustice, and expressions of disappointment increased prosocial intentions (i.e., to help the expresser). The results show that emotional expressions can help people understand ambiguous social situations by informing attributions that correspond with each emotion’s associated appraisal structures. The findings advance understanding of the ways in which emotional expressions help individuals understand and coordinate social life.

## Introduction

At times we may find ourselves thoroughly disliking a situation in which we don’t know what is happening. Unfortunately, social situations are often ambiguous; it may be unclear what others have done, or what they will do next. Such ambiguity can make people uncertain, a state they are motivated to reduce through sense-making (Kagan, 1972; Wong and Weiner, 1981; Heine et al., 2006; Rutjens et al., 2012). These sense-making processes, in turn, are closely related to the experience of emotions. As Frijda (1988, p. 349) put it, “emotions arise in response to the meaning structures of given situations; different emotions arise in response to different meaning structures."

Our own emotions can help us make sense of situations. For example, appraisal theory states that our emotions are accompanied by inferences about the situation, or environment, we are in (Smith and Ellsworth, 1985; Frijda, 1986, 1988; Scherer, 2001; Scherer and Ellsworth, 2003; Clore and Ortony, 2008). Moreover, the emotional expressions^1^ of other people can impact our sense-making processes, by providing information to those people observing their expression (Keltner and Haidt, 1999; Van Kleef, 2009; Van Kleef et al., 2010; Hareli and Hess, 2012).

Imagine a teacher in elementary school who walks into the schoolyard to find a boy crying angrily, or a jury which hears the tale of a defendant who looks away guiltily. Regardless of *what* the teacher or the jury members infer, it seems plausible that they will also use the emotion expressed by the boy or the defendant to inform their judgments of responsibility—that is, to ascribe agency. In the current research, we approach the theoretical relationship between emotional expressions and sense-making. We do so by examining whether emotional expressions can help people resolve ambiguity regarding the causes of events by providing information about agency – that is, who are responsible for a certain state of affairs.

### Ascribing Meaning to Social Events: Attribution and Appraisal

One common way in which people give meaning to their social environment is by analyzing the causes of events. The role that others’ emotional expressions play in this process has been researched in two research traditions: attribution theory and theory on emotion as information. Attribution theory (Weiner, 1985, 2014) holds that people make sense of situations on three dimensions: whether an event is controllable or uncontrollable, internally caused (i.e., by him or herself) or externally caused (i.e., by others or by situational factors), and stable or unstable over time (Weiner, 1985). People attribute causes to own and others’ emotions using a laypersons’ theory (Hareli, 2014), making attribution theory a type of appraisal theory (Weiner, 2014). Because people attribute causes to others’ emotions, attribution theory extends the idea of appraisal to the interpersonal domain (Weiner, 2014).

Recent theorizing on emotion as information has focused on the process by which people respond to the emotions of others. Emotions as social information (EASI) theory (Van Kleef, 2009; Van Kleef et al., 2010, 2011) holds that people who observe an emotional expression may respond to it based on inferential processes and/or affective reactions. Observers may make two types of inferences about the emotion of another person (de Melo et al., 2014). A reverse appraisal involves inferring which appraisal the person experiencing the emotion must have made (Hareli and Hess, 2012), a social appraisal involves inferring aspects of the social situation which then trigger appraisals and emotions in the observer (Manstead and Fischer, 2001).

### Inferences from Others’ Emotions

Attribution theory and emotion as information theory have yielded a substantial body of research on inferences regarding others’ emotional expressions. People may use expressions of emotion to infer the cooperativeness (Van Doorn et al., 2012) and level of risk (e.g., Sorce et al., 1985; Parkinson and Simons, 2009; Parkinson et al., 2012) of the situation in which the expression takes place. Other inferences may include whether the target of the expression performed sufficiently well on a task (Weiner et al., 1979, 1982; Van Doorn et al., 2014). Finally, observers may infer qualities of the person expressing the emotion, such as personality (e.g., Knutson, 1996; Hess et al., 2000; Hareli and Hess, 2010), status (e.g., Tiedens, 2001), moral beliefs (e.g., Horberg et al., 2013), and the likely next behavior of an emotional counterpart in a negotiation (e.g., Van Kleef et al., 2004, 2006; Wubben et al., 2009; de Melo et al., 2014).

In most of the studies above in which social inferences were studied (save for Knutson’s, 1996 work on the inference of stable personality traits), emotion expressions were relatively contextualized, with clear antecedents such as another person’s performance on a test (Weiner et al., 1982), or balloon task (Parkinson et al., 2012), or a bid in a round of negotiations (de Melo et al., 2014). While such contextualization helps draw conclusions regarding the inferences that people make based on an expressed emotion within a specific social context, they say little about social contexts in which an observation is made without an antecedent for the emotion expression being clear. If we are to argue that emotion provides observers with information, it seems important to establish whether its communicative value can be predicted under ambiguity. The aim of this paper, therefore, is to examine whether inferences of agency can reliably be predicted from emotional expressions of anger, regret and disappointment in relatively ambiguous social contexts.

### Anger and Regret as Cues of Agency

People commonly express anger or regret in response to a negative event or outcome (Van Dijk and Zeelenberg, 2002). These emotions differ in the amount of control they imply over the event or outcome. Anger usually involves the attribution of a negative outcome to an external agent, often a person (Averill, 1982; Berkowitz and Harmon-Jones, 2004). It involves the perception that this agent is responsible or blameworthy (Van Dijk and Zeelenberg, 2002; Berkowitz and Harmon-Jones, 2004; Kuppens and Van Mechelen, 2007; Carver and Harmon-Jones, 2009). The experience of regret, in contrast, involves an attribution of self-agency, or internal responsibility, for a negative outcome (Zeelenberg et al., 2000; Van Dijk and Zeelenberg, 2002) implying that the person experiencing the emotion is to be considered responsible or blameworthy. As such, the information which these emotions convey with regards to agency maps onto causality, a central dimension of attribution theory (Weiner, 1985, 2014).

In line with earlier, contextualized differentiations of these emotions with regards to blameworthiness (e.g., de Melo et al., 2014), we expect that in situations in which no clear antecedent for the experience of these emotions is given, a discrepancy in communicated control should lead to different inferences between these emotions with regards to agency. To test this hypothesis, we formulated vignettes describing how another person expressed emotion in an ambiguous situation. We expected that perceivers would associate expressions of anger with attributions of agency to a third person and expressions of regret with attributions of agency to the expressing person.

## Study 1

### Method

#### Participants and Design

Respondents were 70 people from the United States (37 women, age *M* = 35.66, SD = 11.55, range 18–62 years) who participated via Amazon’s Mechanical Turk website (Buhrmester et al., 2011). Participants completed a 10-min survey in exchange for $0.50 USD (a regular rate on the Mechanical Turk website). We asked participants to read one of two different scenarios, in which either anger (*N* = 36) or regret (*N* = 34) was expressed.

#### Materials and Procedure

Participants logged in via the Amazon website, and were redirected to a survey. They read that we were interested in the inferences people make based on minimal information. We prepared a short scenario description, which read: “Suppose you meet a good friend, whom you have not seen for a while. While you are catching up, this friend recalls something that recently happened. Your friend placed an online order for a new cell phone. The phone would be delivered to a store, where your friend could pick it up. As your friend went to the store, a salesperson was there to handle the order. Your friend goes on to tell you the whole story. While telling you what happened, your friend is [getting really angry/feeling very regretful]. Your friend expresses [anger/regret] several times.”

After reading this description, participants completed a questionnaire. First, they indicated attributions with regards to the cause of the emotion expressed by their friend, by ascribing agency to their friend (three items; e.g., “Do you think the emotion of your friend was caused by his or her own behavior?” α = 0.96), another person (three items; e.g., “Do you think the emotion of your friend was caused by another person?” α = 0.97), and the situation (two items; e.g., “Do you think the emotion of your friend was caused by circumstances beyond anyone’s control?” *r* = 0.80, *p* < 0.001).

Please note that because it would not be possible for participants to make inferences regarding a specific antecedent to the expression of emotion, we asked participants to infer agency with regards to the cause of the emotion expressed in the vignettes throughout the studies reported in this paper. Subsequently, participants indicated the extent to which their friend had expressed anger and regret (one item each). All questions were answered on scales ranging from 1 (*not at all*), to 7 (*very much so*).

### Results

Throughout this report, corrected degrees of freedom are reported for *t* tests whenever there was inequality of variances.

#### Manipulation Checks

Participants perceived their friend as more angry in the anger condition (*M* = 6.36, SD = 1.10) than in the regret condition (*M* = 2.82, SD = 1.82), *t*(53.71) = 9.79, *p* < 0.001, *d* = 2.67, *r* = 0.80, and as more regretful in the regret condition (*M* = 6.35, SD = 1.04) than in the anger condition (*M* = 3.83, SD = 1.67), *t*(59.22) = 7.64, *p* < 0.001, *d* = 1.99, *r* = 0.70. We therefore conclude that the emotional expression manipulation was successful.

#### Attributions of Agency^2^

**FIGURE 1:**
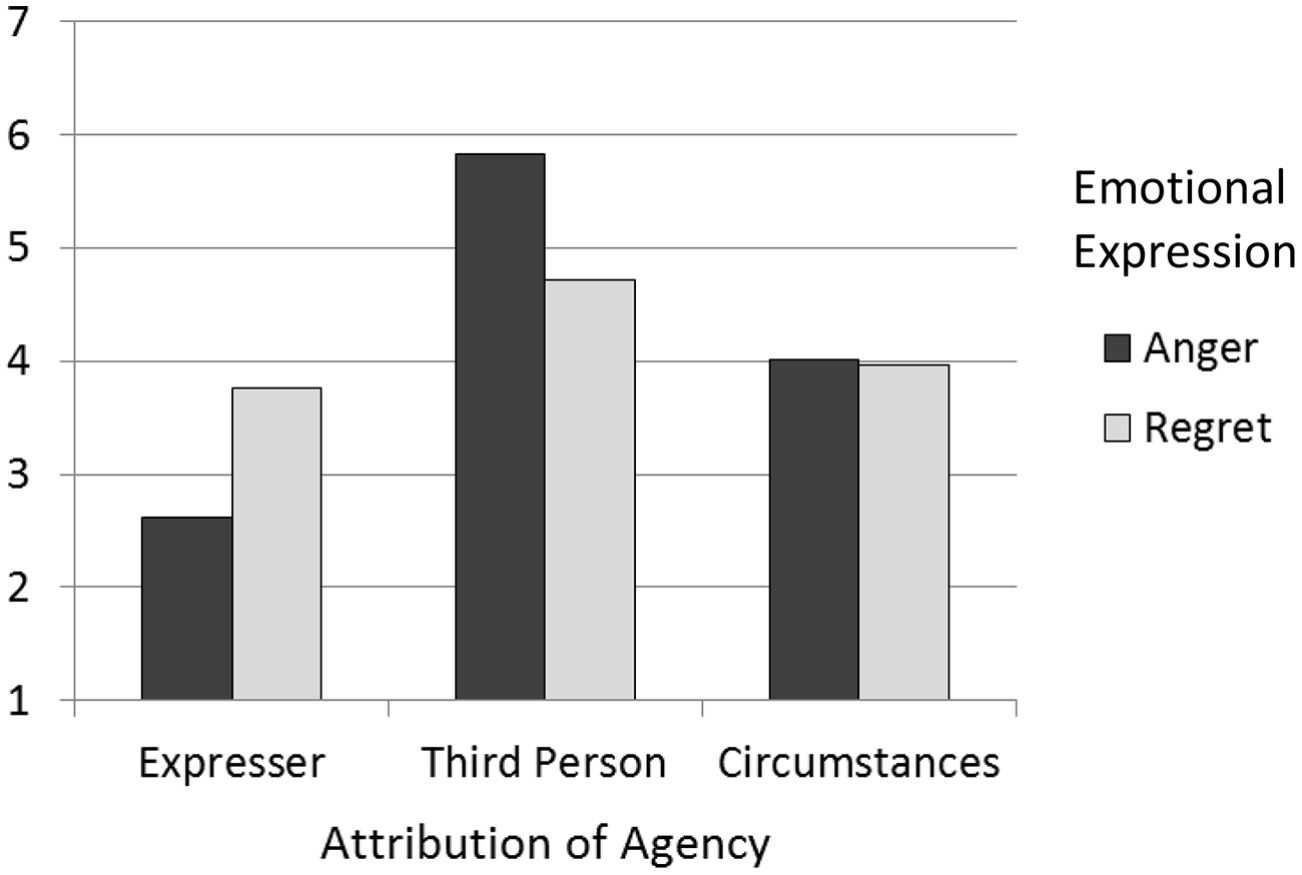
**Effects of emotional expressions on attributions of agency to the expresser, a third person, and circumstances (Study 1)**.

Items for each group of dependent measures were averaged to form scales. We performed *t*-tests between the anger and regret conditions to compare participants’ attributions about the cause of the emotion. As expected, participants attributed more agency to their friend when he or she expressed regret (*M* = 3.76, SD = 1.55) compared to anger (*M* = 2.61, SD = 1.45), *t*(68) = 3.21, *p* = 0.002, *d* = 0.78, *r* = 36. Additionally, participants made less agency attributions to another person when their friend expressed regret (*M* = 4.71, SD = 1.51) compared to anger, *M* = 5.82, SD = 1.03, *t*(57.77) = 3.60, *p* = 0.001, *d* = 0.95, *r* = 0.43. Participants’ attributions of the incident to uncontrollable circumstances did not differ between the anger (*M* = 4.01, SD = 1.43) and regret conditions (*M* = 3.97, SD = 1.39), *t*(68) = 0.83, *p* = 0.41. This pattern of results is visually represented in **Figure 1**.

### Discussion

Findings from Study 1 demonstrate that, as predicted, an expression of anger by a friend led to greater attribution of agency for the expressed emotion to other people and less attribution of agency to the person expressing the emotion, compared to expressions of regret. These findings provide initial evidence that people use others’ emotional expressions as a source of information when attempting to ascribe meaning to ambiguous social situations, in a way that is congruent with the framework of attribution theory.

## Study 2

Based on the findings from Study 1, a clear distinction can be made between anger and regret in terms of the agency that these emotions communicate when they are expressed. However, these differences are relative, in the sense that they were not compared to a control condition in which no emotion expression was mentioned. It is therefore impossible to tell whether the effects were driven by anger, regret, or both. Study 2 included a control condition to allow us to compare the effects of anger and regret to a non-emotional baseline. In addition, we used a scenario with a more severe outcome in Study 2, to examine whether results from Study 1 would generalize.

In addition to these methodological changes, we set out to explore whether the agency effects that we observed in Study 1 would generalize to conceptually related perceptions of responsibility. Anger often involves the appraisal that someone else is to blame for a negative outcome (Kuppens and Van Mechelen, 2007), whereas regret tends to involve the appraisal that oneself is to blame (Zeelenberg et al., 2000). Therefore, it seems plausible that expressions of anger and regret would have comparable effects on perceptions of responsibility and agency. We examined this possibility in Study 2. Finally, we included measures of perceived coping potential and behavioral intentions toward the expresser for exploratory purposes.

### Method

#### Participants and Design

The experiment was completed by 179 participants from the United States (97 female; age M = 35.02, SD = 12.73 years, range 18–67 years), who were again recruited via Amazon’s Mechanical Turk website. As in Study 1, participants completed a 10-min survey in exchange for $0.50 USD. We asked participants to read one of three different scenarios, which again included the anger (N = 62) and regret (N = 58) conditions, as well as a control condition (N = 59) in which no emotion was mentioned.

#### Materials and Procedure

Participants completed the same procedure as in Study 1, but with a different scenario. The scenario used in Study 2 read: “Suppose you meet a good friend, whom you have not seen for a while. While you are catching up, this friend tells you about a recent incident. Your friend was using the car to get to a party at another friend’s place. During the ride, your friend was involved in an accident with another car. Your friend goes on to tell you the whole story. You can see that, while telling you what happened, your friend is [getting really angry/feeling very regretful]. Your friend expresses [anger/regret] several times.” In the control condition, the last two sentences were omitted.

After reading this description, participants completed a questionnaire, consisting of the questions that were also used in Study 1 (emotion caused by friend: α = 0.93; emotion caused by another person: α = 0.95; emotion caused by uncontrollable circumstances: *r* = 0.81, *p* < 0.001) as well as questions regarding the extent to which their friend was responsible for the outcome of the situation (α = 0.92); the extent to which another person was responsible for the outcome of the situation (α = 0.92); the coping ability of the friend (α = 0.64); and participants’ intention to help their friend deal with the situation (α = 0.84). Manipulation checks were the same as in Study 1.

### Results

Means, standard deviations, and specific contrasts for the analyses in Study 2 are reported in **Table 1**.

**Table 1:** Mean ratings on dependent measures in study 2.

	Anger		Regret		No emotion		Omnibus test	
Dependent measure	*M*	SD	*M*	SD	*M*	SD	*F*	*P*
Agency of friend	3.90^a^	1.60	5.56^b^	1.03	4.66^c^	1.15	20.42	<0.001
Agency of others	5.27^a^	1.45	3.36^b^	1.26	4.13^c^	1.36	21.65	<0.001
Agency of circumstances	4.19^a^	1.68	4.08^a^	1.29	4.46^a^	1.55	0.89	0.446
Manipulation check anger	6.63^a^	0.68	2.83^b^	1.63	3.93^c^	1.69	96.24	<0.001
Manipulation check regret	3.32^a^	1.66	6.36^b^	0.99	4.61^c^	1.46	37.99	<0.001
Friend responsible	3.53^a^	1.40	4.62^b^	1.11	3.90^a^	1.14	18.15	<0.001
Other responsible	4.65^a^	1.35	3.39^b^	1.02	3.90^c^	1.06	12.27	<0.001
Friend’s coping potential	3.11^a^	1.01	3.68^b^	0.70	3.29^a^	0.87	6.64	0.002
Intention to help	5.37^a†^	0.79	5.36^a†^	0.75	5.01^b†^	1.04	3.25	0.041

*Means with different superscripts (^a,b,c^) differ at *p* < 0.05. Different superscripts accompanied by ^†^ denote a marginally significant difference (*p* < 0.10).*

#### Manipulation Checks

As expected, analyses of variance (ANOVA) on the manipulation checks showed that participants perceived their friend as more angry in the anger condition than in the other emotion conditions, *F*(2,176) = 117.41, *p* < 0.001, *r* = 0.76. Participants also perceived their friend as more regretful in the regret condition than in the other conditions, *F*(2,176) = 70.54, *p* < 0.001, *r* = 0.67.

#### Attributions of Agency^3^

Analyses of variances with planned contrasts comparing the anger, regret, and no emotion conditions showed that, as in the previous experiment, participants attributed more agency to their friend when their friend expressed regret, compared to when their friend expressed anger, *F*(2,176) = 24.74, *p* < 0.001, *r* = 0.47. Attributions of agency in the control condition fell in between the regret and anger conditions, and differed significantly from both.

As in Study 1, the opposite pattern was observed for attributions of agency to another person in the situation. Participants attributed less agency to another person when their friend expressed regret, compared to when their friend expressed anger, *F*(2,176) = 29.93, *p* < 0.001, *r* = 0.50. Attributions of agency in the control condition again fell in between the regret and anger conditions, and differed significantly from both.

Participants’ attributions of cause to uncontrollable circumstances did not differ significantly between conditions, *F*(2,176) = 0.97, *p* = 0.379.

#### Exploratory Analyses

Differences in means, as indicated in **Table 1**, are based on *post hoc* tests with Tukey correction. As can be seen in this table, participants ascribed different levels of responsibility to their friend, *F*(2,176) = 18.15, *p* < 0.001, *r* = 0.35, and the other person, *F*(2,176) = 12.27, *p* < 0.001, *r* = 0.12, depending on the emotion that was expressed, and they judged their friend’s coping potential in light of the expressed emotion, *F*(2,176) = 6.64, *p* = 0.002, *r* = 0.41. Moreover, participants’ self-reported intention to help their friend cope with the situation also differed, *F*(2,176) = 3,25, *p* = 0.041, *r* = 0.19.

As **Table 1** shows, participants considered their friend most responsible when regret was expressed, and less so when either anger or no emotion were expressed. A similar pattern was found with regards to estimates of the friend’s coping potential. Also, participants considered another person to be most responsible when anger was expressed and least when regret was expressed, with the control condition falling in between. Finally, participants were marginally more likely to help their friend when he or she expressed an emotion compared to when no emotion was expressed.

### Discussion

In Study 2, we replicated and extended the pattern of findings from Experiment 1. Using a different scenario, we found that when their friend was said to express regret, participants again attributed more agency to their friend and less to another person. When their friend was said to express anger, participants attributed less agency to their friend, and more to another person. For both types of attributions, the control condition fell in between the anger and regret conditions, demonstrating that both anger and regret uniquely contribute to the communication of causal properties of the situation. Although including an outcome reduces the ambiguity of the scenario, it is worth noting that the results of the outcome for the person expressing the emotion were not specified beyond it being a negative event, leaving ambiguity with regards to the severity of the consequences.

Exploratory analyses further provided evidence that participants rated their friend as more responsible and better able to cope with the situation following regret than following anger or no emotion. Participants assigned more responsibility to another person in the anger condition, and less in the regret condition, compared to the control condition. Finally, results suggested that participants were somewhat more likely to help their friend deal with the outcome when their friend had expressed anger or regret as opposed to no emotion, although this effect did not reach conventional levels of statistical significance. These results indicate that others’ anger and regret influenced participants’ ascriptions of agency, related inferences regarding responsibility and coping potential, and intentions to help the friend deal with the situation.

## Study 3

In Studies 1 and 2, participants reliably attributed more agency to their friend when (s)he expressed regret, and more agency to another person when their friend expressed anger. In the final study we aimed to extend our findings by examining the effects of expressions of disappointment. In previous research, people who recalled an experience of disappointment were found to appraise the cause of the situation to be due to circumstances beyond their control (Van Dijk and Zeelenberg, 2002). To test whether disappointment leads to similar attributions of agency when it is expressed by another person, we included it as a condition in Study 3. We expected that the disappointment condition would be similar to the control condition with respect to the amount of agency participants would attribute to their friend and another person, but that disappointment would lead to higher levels of attribution of agency to uncontrollable circumstances.

We changed the scenario used in Study 2, so that the friend now described a job interview. An important benefit of using this new scenario was that the expression of disappointment would be a more natural fit to this situation than it would in the scenarios used in the previous studies. As a second benefit, this scenario allowed us to once again leave the outcome ambiguous, replicating Study 2 without the outcome being mentioned explicitly. We also added a measure of perceived injustice. The perception of unfairness or injustice is a typical precursor to the experience of anger (Kuppens et al., 2007). Based on social appraisal accounts (Manstead and Fischer, 2001), a friend’s expression of anger can therefore be expected to lead participants to perceive greater levels of injustice. The measure of perceived injustice was added to explore this possibility.

### Method

#### Participants and Design

We recruited 125 participants from the United States (54 women, age *M* = 30.06, SD = 9.73, range 18–64 years) via Amazon’s Mechanical Turk website. Participants again completed a 10-min survey in exchange for $0.50 USD. We asked participants to read one of four different scenarios, in which the focal person expressed anger (*N* = 33), regret (*N* = 35), disappointment (*N* = 31), or no emotion (*N* = 26).

#### Materials and Procedure

The procedure was identical to that used in Studies 1 and 2, but the scenario was different. It read: “Suppose you meet a good friend, whom you have not spoken to for a while. While you are catching up, this friend tells you about a recent job interview. Your friend applied for a position in a large company. A manager from the personnel department was there to assess whether your friend would be the person for the job. Your friend goes on to tell you the whole story. You can see that, while telling you what happened, your friend is [getting really angry/feeling really disappointed/feeling very regretful]. Your friend expresses [anger/disappointment/regret] several times.” In the control condition, the last two sentences were omitted.

After reading this description, participants once again completed a questionnaire. Besides answering the questions that were also used in Study 2 (emotion caused by friend, α = 0.95; emotion caused by another person, α = 0.96; emotion caused by the situation, *r* = 0.72, *p* < 0.001; coping ability of friend, α = 0.83; friend responsible for outcome, α = 0.88; another person responsible for outcome, α = 0.88; intention to help friend, α = 0.82), participants were further asked to indicate how just they considered the application procedure to have been (four items; e.g., “Do you think the application procedure was fair?” α = 0.87). Finally, manipulation checks were identical to those in the previous studies, except for the fact that one item was added to assess the accuracy of the disappointment manipulation.

### Results

Means, SD, and contrasts for all analyses reported below are shown in **Table 2**.

**Table 2:** Mean ratings on dependent measures in Study 3.

	Anger		Disappointment		Regret		No emotion		Omnibus test	
Dependent Measure	*M*	SD	*M*	SD	*M*	SD	*M*	SD	*F*	*P*
Agency of friend	3.84^a^	1.56	4.31^a,b^	1.50	5.44^c^	1.22	4.72^b^	0.90	8.73	<0.001
Agency of others	5.51^a^	1.29	4.77^b^	1.42	3.54^c^	1.33	4.67^b^	1.19	12.97	<0.001
Agency of circumstances	3.95^a^	1.61	3.61^a^	1.36	3.54^a^	1.35	3.46^a^	1.31	0.73	0.53
Manipulation check anger	6.52^a^	.90	3.35^b^	1.89	3.09^b^	1.46	3.62^b^	1.39	39.86	<0.001
Manipulation check disappointment	5.82^a^	1.31	6.55^c^	0.93	5.31^a,b^	1.45	4.81^b^	1.94	7.92	<0.001
Manipulation check regret	3.91^a^	1.44	4.97^b^	1.54	6.54^c^	0.98	3.85^a^	1.64	26.44	<0.001
Friend responsible	3.12^a^	1.39	3.73^a,b^	1.29	4.27^b^	1.16	3.71^a,b^	0.92	5.08	0.002
Other responsible	4.85^a^	1.10	3.77^b,c^	1.13	3.20^b^	1.35	4.11^a,c^	0.87	12.22	<0.001
Friend’s coping potential	3.85^a†^	1.37	4.61^b†^	1.25	4.66^a,b^	1.32	4.62^b†^	0.86	3.25	0.024
Intention to help	5.33^a,b^	0.82	5.63^a^	0.65	5.53^a,b^	0.75	5.09^b^	0.81	2.83	0.041
Procedural justice	3.87^a†^	1.37	4.76^b^	0.86	4.78^b^	0.98	4.56^b†^	0.93	5.28	0.002

*Means with different superscripts (^a,b,c,d^) differ at *p* < 0.05. Different superscripts accompanied by ^†^ denote a marginally significant difference (*p* < 0.10).*

#### Manipulation Checks

Analyses of variance on the emotion manipulation checks showed that participants perceived their friend as more angry in the anger condition than in the other emotion conditions, *F*(3,121) = 39.86, *p* < 0.001, *r* = 0.71. Participants also perceived their friend as more regretful in the regret condition than in the other conditions, *F*(3,121) = 26.44, *p* < 0.001, *r* = 0.63. Finally, participants perceived their friend as more disappointed in the disappointment condition than in the other conditions, *F*(3,121) = 7.92, *p* < 0.001, *r* = 0.41.

#### Attributions of Agency^4^

Analyses of variance revealed a significant effect of emotion on attributions of agency to the friend, *F*(3,121) = 8.73, *p* < 0.001. As can be seen in **Table 2**, attributions of agency to the friend were higher in the regret condition than in the anger condition. Levels of agency attribution in the disappointment and control conditions fell in between those in the regret and anger conditions, and did not differ from each other.

As in Study 2, the opposite pattern was observed for attributions of agency to another person in the situation, *F*(3,121) = 12.97, *p* < 0.001, *r* = 0.49. Participants attributed less agency to another person when their friend expressed regret, compared to when their friend expressed anger. In the disappointment and control conditions, such attributions again fell in between the regret and anger conditions, and did not deviate from one another.

No effects were found on attributions of agency to uncontrollable circumstances, *F*(3,121) = 0.73, *p* = 0.534. Contrary to our expectation regarding the communicative function of disappointment, participants’ attribution of agency to uncontrollable circumstances did not differ between the disappointment and control conditions.

#### Exploratory Analyses

Differences in means, as indicated in **Table 2**, are based on post-hoc tests with Tukey correction. Participants ascribed different levels of responsibility to their friend, *F*(3,121) = 5.08, *p* = 0.002, *r* = 0.33, and to the other person, *F*(3,121) = 12.22, *p* < 0.001, *r* = 0.48, depending on the emotion expressed, and they also interpreted the friend’s coping potential in light of the emotion expressed, *F*(3,121) = 3.25, *p* = 0.024, *r* = 0.27. Participants’ self-reported intention to help their friend cope with the situation also differed between conditions, *F*(3,121) = 2.83, *p* = 0.041, *r* = 0.26. Finally, participants differed in the extent to which they considered the application procedure to have been just, *F*(3,121) = 5.28, *p* = 0.002, *r* = 0.34.

As **Table 2** shows, participants judged their friend as less responsible in the anger condition than in the regret condition, and they considered their friend’s coping potential to be lower in the anger condition than in the other conditions. They also considered someone else’s responsibility to be higher in the anger condition, compared to the other conditions. Interestingly, participants indicated greater intentions to help their friend deal with the situation when their friend expressed disappointment, compared to when no emotion was expressed. Finally, participants considered the application procedure to have been less just in the anger condition compared to the other conditions.

### Discussion

Results from Study 3 replicate the pattern of agency attributions in the former two studies with a different scenario. We also replicated findings from Study 2 with regards to the inferred responsibility of friend and others and the friend’s perceived coping ability. Moreover, participants considered the application procedure to have been less just in the anger condition, compared to the other conditions, providing additional evidence that the emotions expressed by the friend can (in the absence of other information) inform participants’ assessment of the situation. Although a friend who expressed disappointment regarding his or her outcomes did not lead participants to attribute more agencies to uncontrollable circumstances, disappointment did increase intentions to help the friend cope with the situation, compared to when no emotion was expressed.

## General Discussion

Integrating theorizing on attribution (Weiner, 1985), social appraisal (Manstead and Fischer, 2001), and the use of emotion as social information (Van Kleef, 2009), we conducted three scenario studies to examine how emotional expressions influence attributions of agency and responsibility in ambiguous contexts. We found that expressions of regret about a particular state of affairs led perceivers to attribute greater agency and responsibility for the situation to the expresser, whereas expressions of anger resulted in greater attributions of agency and responsibility to a third person. These studies replicate effects found in previous research in which the expressed emotions had clear contextual antecedents (e.g., Van Kleef et al., 2004, 2006; de Melo et al., 2014). We also found that expressions of anger were interpreted as a sign of injustice, and that expressions of disappointment increased tendencies to help the expresser.

These results show that even when there is no clear antecedent to the expression of emotion by another person, people’s inferences regarding the agency of the expresser and others correspond to the appraisal structures associated with the emotions. These results indicate that inferences that people make regarding the person expressing the emotion don’t necessarily rely on a preceding outcome being known, and make an account of emotions as social information less sensitive to context and, therefore, stronger. Our results are novel in that under conditions of ambiguity, inferences of agency are a markedly more dynamic class of social inferences than inferences of stable personality traits (Knutson, 1996). Accordingly, one promising avenue for further research is to determine whether effects of emotional expressions on inferences about the situation in which the emotion is expressed, qualities of the expressing person, and qualities of the person to whom the expression is directed can be similarly decontextualized.

People may use social appraisal (Manstead and Fischer, 2001), reverse appraisal (Hareli and Hess, 2012), or a combination thereof to make inferences based on others’ emotions (de Melo et al., 2014). When inferences result from one, or both, of these processes is currently unclear. In the current studies, social appraisals regarding agency and responsibility could reliably be made, despite a lack of a contextual antecedent of (cause of) the emotion expression. Because no antecedent event was described as a trigger for the emotion that was being expressed, however, it seems less likely that our participants engaged in the reconstruction of the appraisals of the person expressing an emotion (i.e., reverse appraisal; de Melo et al., 2014). The information needed to do so was simply not available to them. A promising line for future research could be to investigate which information observers use in order to reliably reconstruct the appraisals underlying an expression of emotion which they observe.

Interestingly, Study 3 revealed no evidence that the expression of disappointment influences attributions of agency and responsibility. It thus seems that the link between disappointment and situational agency, which was found in previous research (e.g., Van Dijk and Zeelenberg, 2002), did not translate to social appraisals, perhaps because the experience of disappointment often results in inaction (Zeelenberg et al., 2000). Understanding why someone does *not* act may be less important than understanding why someone does act, and may hence have less of an impact on the social appraisals (Manstead and Fischer, 2001) that observers themselves are likely to make of the situation. Interestingly, the expression of disappointment did increase self-reported intentions to help the expresser. This finding is in line with research on prosocial behavior (Van Doorn et al., 2015) and negotiation (Van Kleef et al., 2006; Lelieveld et al., 2013), which also yielded evidence that expressions of disappointment can elicit cooperative behavior. Whether disappointment reliably yields a social appraisal that someone should be helped, however, is a question for further research.

In interpreting the current findings, a number of potential limitations of our approach must be considered. First, we presented participants with hypothetical scenarios, rendering it unclear at this point to what extent the current findings generalize to actual situations. Although similar methodology was used previously in research on attribution processes (e.g., Weiner, 1985), it will be important to replicate the current effects in more dynamic, real-world situations (Parkinson and Manstead, 1993). A second potential limitation of the present studies concerns the reliance on verbal descriptions of emotional expressions. Emotions may be expressed in various ways, including via facial displays, vocal cues, bodily postures, verbal expressions, and/or symbols such as emoticons. Despite the obvious qualitative differences between these various expressive modalities, there is increasing evidence that the interpersonal effects of emotional expressions are functionally equivalent in that the direction (but not necessarily the magnitude) of their effects on other individuals is the same across expressive channels (Van Kleef et al., 2011).

This observation is consistent with a social-functional approach to emotions (Darwin, 1872; Parkinson, 1996; Keltner and Haidt, 1999; Van Kleef, 2009; Fischer and Manstead, in press). For instance, a basic assumption underlying EASI theory is that individuals turn to each other’s emotional expressions to make sense of ambiguous (social) situations, and that such disambiguating information can be gleaned from verbal as well as non-verbal expressions (Van Kleef et al., 2011). In line with this “functional equivalence hypothesis,” recent studies on the role of emotional expressions in persuasion and conformity showed that effects were similar regardless of whether emotions were expressed in words, through facial displays, via emoticons, or via a combination of facial, vocal, and postural cues (Heerdink et al., 2013; Van Kleef et al., 2015). In light of this evidence, and earlier work by Knutson (1996) who found effects of facial expressions of emotion on inferences regarding the personality of the expressing person, we assume that we would have found similar effects in the current studies if we had manipulated emotional expressions using non-verbal cues. Clearly, however, future research is needed to substantiate this assumption.

Awaiting further investigations, we conclude that people use others’ emotional expressions as a source of information when attempting to make sense of social situations. More specifically, individuals use the emotional expressions of others to arrive at inferences regarding others’ agency and responsibility for a current state of affairs, which correspond with the appraisal structures associated with the emotions. These findings contribute to a growing body of research that speaks to the ways in which individuals draw on conceptual emotion knowledge to interpret the emotional expressions of others. Such inferential processes play an important role in the construal and navigation of social life.

## Author Contributions

EvD, GvK, and JvdP developed the study hypotheses and designs, EvD collected and analyzed the data, EvD drafted the paper, GvK and JvdP provided feedback and made revisions, and GvK prepared the paper for submission. EvD revised the manuscript following reviews.

## Conflict of Interest Statement

The authors declare that the research was conducted in the absence of any commercial or financial relationships that could be construed as a potential conflict of interest.

## Footnotes

1 The ‘emotional expression’ is often used to denote *facial* expressions of emotion. In this paper, we use the term to refer to the expression of emotion through words. Whether or not the effects observed in this paper also occur when facial expressions are being used is a question that is beyond the scope of this paper. We return to this issue in the General Discussion.

2 ^2^In order to examine whether the attributions of agency that participants made following the various emotions were affected by gender, we re-ran the analyses reported above, this time including participant gender as an exploratory factor (there were at least 16 participants in each cell of the 2 × 2 design). No effects of gender were observed on attributions of agency to the friend, to other people, or to circumstances, all *F* values <1.17, all *p* values >0.28.

3 ^3^To examine effects of gender, we re-ran the analyses for all attributions of agency. All cells in the design included a minimum of 22 participants. No effects of gender (main or interaction) were observed on attributions of agency to the friend, other people, or circumstances, all *F* values <1.34, all *p* values >0.25.

4 ^4^To examine effects of gender, we again re-ran the analyses for all causal attributions. All cells in the design included a minimum of 10 participants. No effects of gender (main or interaction) were observed on attributions of agency to the friend, other people, or circumstances, all *F* values <1.73, all *p* values >0.16.

## References

[B1] Averill J. R. (1982). *Anger and Aggression*. New York, NY: Springer 10.1007/978-1-4612-5743-1

[B2] Berkowitz L. Harmon-Jones E. (2004). Toward an understanding of the determinants of anger. *Emotion* 4 107–130. 10.1037/1528-3542.4.2.107 15222847

[B3] Buhrmester M. Kwang T. Gosling S. D. (2011). Amazon’s mechanical turk: a new source of inexpensive, yet high quality data? *Perspect. Psychol. Sci.* 6 3–5. 10.1177/1745691610393980 26162106

[B4] Carver C. S. Harmon-Jones E. (2009). Anger is an approach-related affect: evidence and implications. *Psychol. Bull.* 135 183–204. 10.1037/a0013965 19254075

[B5] Clore G. L. Ortony A. (2008). “Appraisal theories: how cognition shapes affect into emotion,” in *Handbook of Emotions* 3rd Edn eds Lewis M. Haviland-Jones J. M. Feldmann Barret L. (New York, NY: Guilford Press) 628–644.

[B6] Darwin C. (1872). *The Expression of the Emotions in Man and Animals* 3rd Edn. London: HarperCollins 10.1037/10001-000

[B7] de Melo C. M. Carnevale P. J. Read S. J. Gratch J. (2014). Reading people’s minds from emotion expressions in interdependent decision making. *J. Pers. Soc. Psychol.* 106 73–88. 10.1037/a0034251 24079297

[B8] Fischer A. H. Manstead A. S. R. (in press). “Social functions of emotion and emotion regulation,” in *Handbook of Emotion* 4th Edn eds Lewis M. Haviland J. Feldman Barrett L. (New York, NY: Guilford).

[B9] Frijda N. H. (1986). *The Emotions*. Cambridge: Cambridge University Press.

[B10] Frijda N. H. (1988). The laws of emotion. *Am. Psychol.* 43 349–358. 10.1037/0003-066X.43.5.349 3389582

[B11] Hareli S. (2014). Making sense of the social world and influencing it by using a naïve attribution theory of emotions. *Emot. Rev.* 6 336–343. 10.1177/1754073914534501

[B12] Hareli S. Hess U. (2010). What emotional reactions can tell us about the nature of others: an appraisal perspective on person perception. *Cogn. Emot.* 24 128–140. 10.1080/02699930802613828

[B13] Hareli S. Hess U. (2012). The social signal value of emotions. *Cogn. Emot.* 26 385–389. 10.1080/02699931.2012.665029 22471847

[B14] Heerdink M. W. Van Kleef G. A. Homan A. C. Fischer A. H. (2013). On the social influence of emotions in groups: interpersonal effects of anger and happiness on conformity versus deviance. *J. Pers. Soc. Psychol.* 105 262–284. 10.1037/a0033362 23773049

[B15] Heine S. J. Proulx T. Vohs K. D. (2006). Meaning maintenance model: on the coherence of social motivations. *Pers. Soc. Psychol. Rev.* 10 88–110. 10.1207/s15327957pspr1002_1 16768649

[B16] Hess U. Blairy S. Kleck R. E. (2000). The influence of facial emotion displays, gender and ethnicity on judgments of dominance and affiliation. *J. Nonverbal Behav.* 24 265–283. 10.1023/A:1006623213355

[B17] Horberg E. J. Kraus M. W. Keltner D. (2013). Pride displays communicate self-interest and support for meritocracy. *J. Pers. Soc. Psychol.* 105 24–37. 10.1037/a0032849 23713701

[B18] Kagan J. (1972). Motives and development. *J. Pers. Soc. Psychol.* 22 51–62. 10.1037/h0032356 5013358

[B19] Keltner D. Haidt J. (1999). Social functions of emotions at four levels of analysis. *Cogn. Emot.* 13 505–521. 10.1080/026999399379168

[B20] Knutson B. (1996). Facial expressions of emotion influence interpersonal trait inferences. *J. Nonverbal Behav.* 20 165–182. 10.1007/BF02281954

[B21] Kuppens P. Van Mechelen I. (2007). Interactional appraisal models for the anger appraisals of threatened self-esteem, other-blame, and frustration. *Cogn. Emot.* 21 56–77. 10.1080/02699930600859219

[B22] Kuppens P. Van Mechelen I. Smits D. J. M. De Boeck P. Ceulemans E. (2007). Individual differences in patterns of appraisal and anger experience. *Cogn. Emot.* 21 689–713. 10.1080/02699930600859219

[B23] Lelieveld G.-J. Van Dijk E. Van Beest I. Van Kleef G. A. (2013). Does communicating disappointment in negotiations help or hurt? Solving an apparent inconsistency in the social-functional approach to emotions. *J. Pers. Soc. Psychol.* 105 605–620. 10.1037/a0033345 23773043

[B24] Manstead A. S. R. Fischer A. H. (2001). “Social appraisal: the world as object of and influence on appraisal processes,” in *Appraisal Processes in Emotion: Theory, Methods, Research* eds Scherer K. R. Schorr A. Johnstone T. (Oxford: Oxford University Press) 221–252.

[B25] Parkinson B. (1996). Emotions are social. *Br. J. Psychol.* 87 663–683. 10.1111/j.2044-8295.1996.tb02615.x 8962482

[B26] Parkinson B. Manstead A. S. R. (1993). Making sense of emotion in stories and social life. *Cogn. Emot.* 7 295–323. 10.1080/02699939308409191

[B27] Parkinson B. Phiri N. Simons G. (2012). Bursting with anxiety: adult social referencing in an interpersonal Balloon Analogue Risk Task (BART). *Emotion* 12 817–826. 10.1037/a0026434 22251046

[B28] Parkinson B. Simons G. (2009). Affecting others: social appraisal and emotion contagion in everyday decision making. *Pers. Soc. Psychol. Bull.* 35 1071–1084. 10.1016/j.jesp.2009.04.010 19474455

[B29] Rutjens B. T. Van der Pligt J. Van Harreveld F. (2012). “Regulating psychological threat: the motivational consequences of threatening contexts,” in *Restoring Civil Societies: The Psychology of Intervention and Engagement following Crisis* eds Jonas K. J. Morton T. (Chichester: Wiley Blackwell).

[B30] Scherer K. R. (2001). “Appraisal considered as a process of multi-level sequential checking,” in *Appraisal Processes in Emotion: Theory, Methods, Research* eds Scherer K. R. Schorr A. Johnstone T. (Oxford: Oxford University Press) 92–120.

[B31] Scherer K. R. Ellsworth P. C. (2003). “Appraisal processes in emotion,” in *Handbook of Affective Sciences* eds Davidson R. J. Scherer K. R. Goldsmith H. H. (Oxford: Oxford University Press).

[B32] Smith C. A. Ellsworth P. C. (1985). Patterns of cognitive appraisal in emotion. *J. Pers. Soc. Psychol.* 48 813–838. 10.1037/0022-3514.48.4.813 3886875

[B33] Sorce J. F. Emde R. N. Campos J. J. Klinnert M. D. (1985). Maternal emotional signaling: its effect on the visual cliff behavior of 1-year-olds. *Dev. Psychol.* 21 195–200. 10.1037/0012-1649.21.1.195

[B34] Tiedens L. Z. (2001). Anger and advancement versus sadness and subjugation: the effect of negative emotion expressions on social status conferral. *J. Pers. Soc. Psychol.* 80 86–94. 10.1037//0022-3514.80.1.86 11195894

[B35] Van Dijk W. W. Zeelenberg M. (2002). Investigating the appraisal patterns of regret and disappointment. *Motiv. Emot.* 36 321–331. 10.1023/A:1022823221146

[B36] Van Doorn E. A. Heerdink M. W. Van Kleef G. A. (2012). Emotion and the construal of social situations: inferences of cooperation versus competition from expressions of anger, happiness and disappointment. *Cogn. Emot.* 26 442–461. 10.1080/02699931.2011.648174 22471851

[B37] Van Doorn E. A. Van Kleef G. A. Van der Pligt J. (2014). How instructors’ emotional expressions shape students’ learning performance: the roles of anger, happiness, and regulatory focus. *J. Exp. Psychol. Gen.* 143 980–984. 10.1037/a0035226 24364685

[B38] Van Doorn E. A. Van Kleef G. A. Van der Pligt J. (2015). How emotional expressions shape prosocial behavior: interpersonal effects of anger and disappointment on compliance with requests. *Motiv. Emot.* 39 128–141. 10.1007/s11031-014-9421-6

[B39] Van Kleef G. A. (2009). How emotions regulate social life: the emotions as social information (EASI) model. *Curr. Dir. Psychol. Sci.* 18 184–188. 10.1111/j.1467-8721.2009.01633.x

[B40] Van Kleef G. A. De Dreu C. K. Manstead A. S. (2004). The interpersonal effects of anger and happiness in negotiations. *J. Pers. Soc. Psychol.* 86 57–76. 10.1037/0022-3514.86.1.57 14717628

[B41] Van Kleef G. A. De Dreu C. K. W. Manstead A. S. R. (2006). Supplication and appeasement in conflict and negotiation: the interpersonal effects of disappointment, worry, guilt, and regret. *J. Pers. Soc. Psychol.* 91 124–142. 10.1037/0022-3514.91.1.124 16834484

[B42] Van Kleef G. A. De Dreu C. K. W. Manstead A. S. R. (2010). An interpersonal approach to emotion in social decision making: the emotions as social information model. *Adv. Exp. Soc. Psychol.* 42 45–96. 10.1016/S0065-2601(10)42002-X

[B43] Van Kleef G. A. Van den Berg H. Heerdink M. W. (2015). The persuasive power of emotions: effects of emotional expressions on attitude formation and change. *J. Appl. Psychol.* 100 1124–1142. 10.1037/apl0000003 25402955

[B44] Van Kleef G. A. Van Doorn E. A. Heerdink M. W. Koning L. F. (2011). Emotion is for influence. *Eur. Rev. Soc. Psychol.* 22 111–163. 10.1080/10463283.2011.627192

[B45] Weiner B. (1985). An attribution theory of achievement motivation and emotion. *Psychol. Rev.* 92 548–573. 10.1037/0033-295X.92.4.548 3903815

[B46] Weiner B. (2014). The attribution approach to emotion and motivation: history, hypotheses, home runs, headaches/heartaches. *Emot. Rev.* 6 353–361. 10.1177/1754073914534502

[B47] Weiner B. Graham S. Stern P. Lawson M. E. (1982). Using affective cues to infer causal thoughts. *Dev. Psychol.* 18 278–286. 10.1037//0012-1649.18.2.278

[B48] Weiner B. Russell D. Lerman D. (1979). The cognition-emotion process in achievement-related contexts. *J. Pers. Soc. Psychol.* 37 1211–1220. 10.1037/0022-3514.37.7.1211

[B49] Wong P. T. Weiner B. (1981). When people ask “why” questions, and the heuristics of attributional search. *J. Pers. Soc. Psychol.* 40 650–663. 10.1037/0022-3514.40.4.650

[B50] Wubben M. J. J. De Cremer D. D. Van Dijk E. (2009). How emotion communication guides reciprocity: establishing cooperation through disappointment and anger. *J. Exp. Soc. Psychol.* 45 987–990. 10.1016/j.jesp.2009.04.010

[B51] Zeelenberg M. Van Dijk W. W. Manstead A. S. R. Van der Pligt J. (2000). On bad decisions and disconfirmed expectancies: the psychology of regret and disappointment. *Cogn. Emot.* 14 521–541. 10.1080/026999300402781

